# Causal Analysis of Learning Performance Based on Bayesian Network and Mutual Information

**DOI:** 10.3390/e21111102

**Published:** 2019-11-11

**Authors:** Jing Chen, Jun Feng, Jingzhao Hu, Xia Sun

**Affiliations:** School of Information Science and Technology, Northwest University, Xi’an 710127, China; jamiechen@stumail.nwu.edu.cn (J.C.); hujingzhao@stumail.nwu.edu.cn (J.H.)

**Keywords:** causal analysis, Bayesian network, mutual information, learning performance prediction, personalized interventions

## Abstract

Over the past few years, online learning has exploded in popularity due to the potentially unlimited enrollment, lack of geographical limitations, and free accessibility of many courses. However, learners are prone to have poor performance due to the unconstrained learning environment, lack of academic pressure, and low interactivity. Personalized intervention design with the learners’ background and learning behavior factors in mind may improve the learners’ performance. Causality strictly distinguishes cause from outcome factors and plays an irreplaceable role in designing guiding interventions. The goal of this paper is to construct a Bayesian network to make causal analysis and then provide personalized interventions for different learners to improve learning. This paper first constructs a Bayesian network based on background and learning behavior factors, combining expert knowledge and a structure learning algorithm. Then the important factors in the constructed network are selected using mutual information based on entropy. At last, we identify learners with poor performance using inference and propose personalized interventions, which may help with successful applications in education. Experimental results verify the effectiveness of the proposed method and demonstrate the impact of factors on learning performance.

## 1. Introduction

In the past few years, online learning has been increasingly taking center stage outside the classroom due to potentially unlimited enrollment, lack of geographical limitations, and free access for many courses [1]. Online courses have attracted substantially billions of learners [2]. Considering the large number of learners, one major concern that should not be neglected is whether the learning performance is effective. The grade distributions of courses are heavily skewed, with only 10% of learners achieving a perfect grade. Many learners have poor performance, achieving a low grade or even zero [3].

There are two methods to improve learning performance, a teaching-oriented method and learner-oriented method. The teaching-oriented method focuses on lecture design and improvement, such as improving lecture content [4], designing tests for lectures [5] and online game-based teaching [6]. This method helps to provide high-quality educational resources and diverse teaching methods. However, it lacks personalized interventions to improve learning performance. The learner-oriented method focuses on making effective and personalized interventions [7,8], which will help to accelerate the growth of learners explicitly. The intervened learners will be given additional opportunities to track and master their learning of concepts, which will improve the their performance.

Traditional learning provides a face-to-face environment where teachers can provide timely feedback and interventions. However, in an online learning environment, it is unrealistic to expect teachers to fully track learners’ learning and to provide timely personalized interventions [9]. Fortunately, learners generate plenty of data when interacting with online learning platforms. These data include learning factors and learning performance which can be collected automatically by the platforms. Thus, in this new learning environment, we need machine learning to analyze the relationship between learning factors and learning performance.

Learning performance has different definitions in different learning environments. Many criteria can measure learning performance, such as the completion rate of a course, grade, the likelihood of getting a certificate, added knowledge, and skill building, depending on the course content and the learner’s nature [10]. A course grade is a numerical summary of a selected course that shows how well a learner understands and applies the knowledge conveyed in the course [11]. Moreover, it is commonly used in learning performance prediction in an online learning environment [12,13,14,15]. Therefore, in this paper, grade is utilized as an objective indicator of learning performance.

Learning factor plays a significant role in improving learning performance. Once the factors possibly leading to poor performance are identified, we can analyze the underlying reason and provide corresponding interventions. Factors in this paper can be categorized into two groups: background factors and learning behavior factors. Background factors include education (highest level of education completed), age (age brackets), motivation (reason for taking the course), learning type (learning type of learners, e.g., positive), and expected learning hours (range of expected learning hours per week), which have an impact on learning behaviors and learning performance. Learning behavior factors include views (whether views are equal to or more than 50% of the content modules), assignments (completed assingments equal to or more than three), completion (percent of content modules a learner has completed), forum posts (total number of posts in discussion forums), events (number of distinct interaction events with the course), and active days (number of active days with one or more events), which have an impact on learning performance. A detailed description of factors is shown in Table 1 and Table 2.

Many studies have focused on correlations [16,17,18] between factors and learning performance. A correlation is a relationship that can be observed between factors that appear to be related [19]. Correlation analysis is a statistical method used to evaluate the degree of association between two numerically measured factors [20]. Sometimes, two strongly correlated factors may not have any causal relationship and a correlation between two factors is completely symmetrical.

Unlike correlation, a causal relationship strictly distinguishes cause from outcome factors, which is asymmetrical. Once you find out the cause leading to a certain outcome, you may make changes to meet your needs. Causal analysis is more reliable than correlation analysis and plays an irreplaceable role in design guiding interventions [21]. Causal analysis has been applied to many fields successfully, such as industry [22], medical decision making [23], and environmental modeling [24]. Causal analysis of learning performance is also an important direction in education [25]. A few studies conduct causal analysis between factors and learning performance [26,27]. That may support educators to make effective interventions, leading to successful applications such as intelligent tutoring, personalized recommendations, and learning evaluations.

There are three main kinds of methods to make causal analyses, randomized controlled trials, quasi-experimental designs, and probabilistic graphical models. A randomized control trial is a trial in which subjects are randomly assigned to one of two groups: the experimental group receiving the intervention that is being tested, and the comparison or control group receiving an alternative treatment [28]. However, this requires a deep understanding and high control capability of the experimental data. A quasi-experiment can be used to empirically estimate the causal impact of an intervention on a target subject without random assignments. Although quasi-experimental designs are recommended for educational causal analysis, their empirical justification is inferior to that of the standard experiment [29]. Cook et. al demonstrated that quasi-experiments regularly failed to reproduce experimental results unless the assignment mechanism was completely known or extensively and reliably measured [30]. Due to the massively collected educational data, quite a lot of studies have adopted machine learning methods to preform causal analysis, especially in the online learning environment. The probabilistic graphical model is one of the most used machine learning methods in causal analysis, which has been successfully applied in many fields. The benefits of the probabilistic graphical model are involving uncertainty in the modelling, resulting in less sensitivity to noise data.

As a typical probabilistic graphical model, a Bayesian network is a powerful tool for modeling the causal relationships among factors and can easily complete inference [31]. It can implement learning performance prediction and helps us to explore factors resulting in poor learning performance. Thus, we adopt a Bayesian network to make a causal analysis of learning performance.

The goal of this paper is to complete a causal analysis of learning performance and then provide personalized interventions for learners considering their specific background and learning behavior factors. Our contributions are as follows. This paper first constructs a Bayesian network for causal analysis based on learners’ background and learning behavior factors. Secondly, the important factors in the constructed network are selected using mutual information based on entropy. Thirdly, we identify learners with poor performance using inference and propose personalized interventions based on the selected factors, which may help with successful applications in education.

## 2. Related Work

Causal analysis of learning performance is important for designing interventions. The primary method of causal analysis in learning performance is the randomized control trial (RCT). RCTs have played an important role in determining whether an intervention is having a measurable effect on learning [32]. Bradshaw et al. used RCTs to examine the effects of positive behavioral interventions and supported on student performance. The results demonstrated significant reductions in student suspensions [33]. Nevertheless, RCTs require deep understanding and high control capability of the experimental data. Moreover, it tends to generate simplistic universal rules of cause and effect, and it is inherently descriptive and contributes little to theory [34].

The second method to make causal analysis of learning performance is a quasi-experiment. Lusher et al. exploited a long term quasi-experiment where students alternated between morning and afternoon school blocks every month. The experimental results provided a causal evidence of student performance during double-shift schooling systems, that a precisely estimated drop was found in student performance during afternoon blocks. Although quasi-experiment are an effective method to make causal analysis and have been applied in education frequently, experimental results can be reproduced only if the assignment mechanism is completely known or extensively and reliably measured.

In addition to the two traditional methods, a few studies utilized machine learning methods to perform causal analysis. Wang et al. proposed a causal analysis algorithm by improving the Apriori algorithm to analyze the relationship between learning behaviors and performance, and provided an application direction for a daily inspection system based on the learning behaviors [26]. Ramirez-Arellano et al. proposed a model that described the causal relationships concerning motivations, emotions, cognitive strategies, meta-cognitive strategies, learning strategies, and their impact on learning performance [27].

As a widely used machine learning method for causal analysis, a Bayesian network can demonstrate the causal relationship between factors graphically and can easily complete inference. There are two methods to construct a Bayesian network: expert knowledge, which relies on professional experience; and structure learning, which automatically learns relationships from data. Some of the studies adopted expert knowledge to construct the Bayesian network and then made a causal analysis. Millán et al. constructed student models for first degree equations using a Bayesian network based on expert knowledge. Those models were used to obtain accurate estimations of student’s knowledge on the same concepts and made their analysis [35]. However, only using expert knowledge may ignore some non-remarkable relationships. In addition, experts may have different opinions towards the relationship of the same pair of factors.

Certain studies have utilized structure learning to mine the causal relationship between factors. Millán et al. compared the performance of the student models constructed by expert knowledge and structure learning respectively. The results demonstrated that both models were able to provide reasonable estimations for knowledge variables [36]. The structure learning method relies on a large amount of data to obtain reliable relationships. Thus, we combine the expert knowledge and structure learning to make causal analysis, because the structure learning can be elicited with the help of experts knowledge. In the meanwhile, we need to reduce inconsistencies among experts in the expert knowledge.

A common method to combine expert knowledge and structure learning is to add constraints during structure learning. There are mainly two types of constraints: parameter constraints, which define rules about the probability values inside the local distributions; and structural constraints, which specify arcs, may or may not be included. The authors in [37] proposed an algorithm to learn Bayesian network structure from data and expert knowledge, integrating parameter constraints and structure constraints. Niculescu et al. incorporated parameter constraints into learning process of Bayesian networks, considering domain knowledge that constrains the values among subsets of parameters with a known structure [38]. Perrier et al. utilized structural constraints to reduce the search space when learning structure from data [39]. In our study, the knowledge of connection between cause and effect is easy to be obtained by experts. Thus, we use structure constraints to incorporate expert knowledge during structure learning.

The remainder of the paper is organized as follows. Section 3 describes the proposed framework and detailed methods. Section 4 presents the dataset and the experimental tool used in this paper. Section 5 demonstrates the experimental results and the interventions. The discussion and conclusion are drawn in Section 6 and Section 7.

## 3. Method

### 3.1. Overall Framework

**Figure 1:**
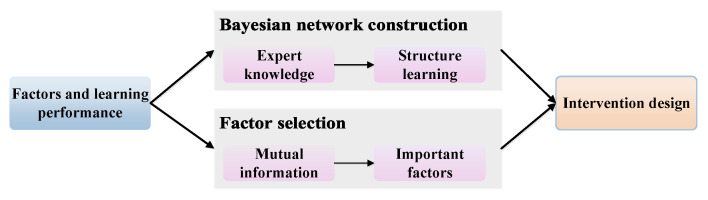
The theoretical framework. The input of the framework is factors and learning performance. The method module includes Bayesian network construction, factor selection, and intervention design.

To achieve our goal, we propose a framework as shown in Figure 1. Factors and learning performance are input into the Bayesian network construction module and factor selection module, to construct the network and identify important factors, respectively. For Bayesian network construction, we first construct an initial Bayesian network from expert knowledge and then use the structure learning method to add some relationships not in the initial network. For factor selection, we use mutual information based on entropy to find important factors towards the target factor. Next, the constructed network and important factors are input into the intervention design module to propose personalized interventions for different learners.

### 3.2. Bayesian Network Construction

In this paper, we construct a Bayesian network (BN) to represent the causal relationships between factors. A BN is comprised of a qualitative part and a quantitative part. The qualitative part is a directed acyclic graph. The factors and their causal relationships are represented as nodes and directed arcs, respectively. The parents of each node are its direct causes. The quantitative part of a BN is its conditional probability tables where local conditional probabilities are mapped into the factors. A conditional probability table specifies the probability of each state of a factor given its parents. Tables for root nodes only contain unconditional probabilities. The BN is represented as a pair (G,P), where *G* is a directed acyclic graph over a set of factors $X=X_{1},X_{2},X_{3},\ldots X_{n}$ and *P* is a joint probability distribution of *X*. *P* can be calculated by (1), multiplying the conditional probabilities of every factor given its parent nodes, under conditional independence assertions.

(1) $$P\left(X_{1},X_{2},\ldots ,X_{n}\right)=\prod_{i=1}^{n}P(X_{i}|Parent\left(X_{i}\right)).$$

The BN not only demonstrates the graphic structure among factors but also measures the relationships among factors quantitatively. Learning performance prediction and personalized intervention design rely on the graph structure and corresponding conditional probability table of each node. When new observations are obtained, such as background and learning behavior factors, the states of those observations are determined. Next, the state probabilities of the target factor, such as learning performance, will be calculated using the probabilistic method.

**Figure 2:**
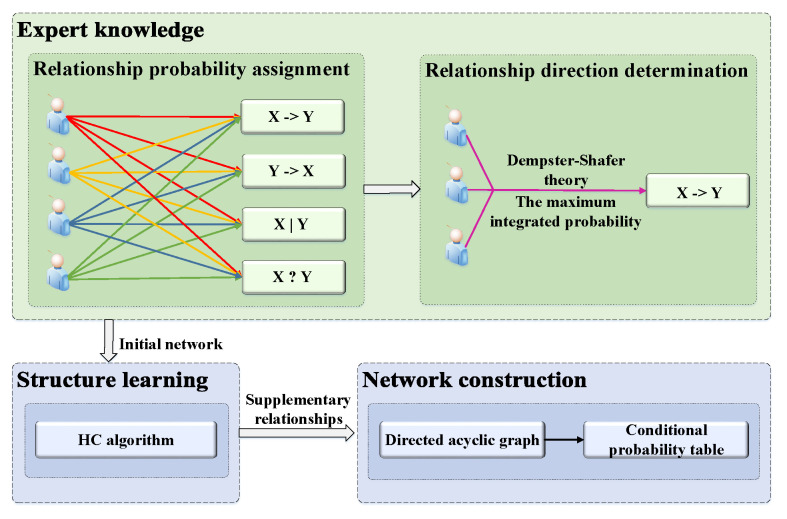
Process of network construction. There are four steps to construct a Bayesian network (BN), including relationship probability assignment, relation direction determination, structure learning, and network construction.

To construct a BN, a directed acyclic graph should be built first, which reflects the causal relationship of the desired factors. Secondly, the conditional probability table for each factor is estimated. There are two methods to construct a directed acyclic graph, using expert knowledge and the structure learning algorithm. The former relies on the experience of experts in education. In this way, some non remarkable causal relationships between factors may be omitted. Using the structure learning algorithm means that the network structure is learned from data. However, this approach needs a large amount of data. Under the condition of a limited amount of data, the graphic network learned from data may not be accurate [22]. Therefore, in this study, we combine those two methods to construct the network. The constructing process is shown in Figure 2.

Step 1. Relationship probability assignment. There are four relationships between each pair of factors. For example, the four possible relationships between factor *X* and factor *Y* are as follows: *X* directly influencing *Y* (X→Y), *Y* directly influencing *X* (Y→X), no relationship between *X* and *Y* (X|Y), and uncertain relationship between *X* and *Y* (X?Y). An odd number of educational experts are requested to assign a probability for each of these four possible relationships; the sum of the four assigned probabilities is equal to one.

Step 2. Relationship direction determination. To reduce inconsistencies among experts, we utilize the Dempster–Shafer theory [22] to integrate the probabilities of the four possible relationships from different experts. The relationship with the maximum value between each pair of factors is adopted to represent the specified relationship. The equations used for integration are as follows:

(2) $$P\left(R\right)=\frac{1}{K}\sum_{R_{1}\cap R_{2}\cdots \cap R_{n}=R}P_{1}\left(R_{1}\right)\cdot P_{2}\left(R_{2}\right)\cdots P_{n}\left(R_{n}\right)$$

(3) $$\begin{array}{ll} K & =1-\sum_{R_{1}\cap R_{2}\cdots \cap R_{n}=\emptyset }P_{1}\left(R_{1}\right)\cdot P_{2}\left(R_{2}\right)\cdots P_{n}\left(R_{n}\right)\\ & =\sum_{R_{1}\cap R_{2}\cdots \cap R_{n}\neq \emptyset }P_{1}\left(R_{1}\right)\cdot P_{2}\left(R_{2}\right)\cdots P_{n}\left(R_{n}\right) \end{array}$$

where P(R) is the integrated probability for each relationship. $P_{n}R_{n}$ is the probability that the $n_{th}$ expert specifies a relationship. *K* is the normalizing factor and 1−K is a measure of the amount of conflict information. The detailed calculation process is shown in Section 5.1. If there exists a cycle in the network, we will remove the edge with the minimum integration probability of the network. That means the most uncertain relationship in a cycle will be removed to guarantee the acyclicity.

Step 3. Structure learning. To avoid ignoring non-obvious and reasonable causal relationships, we utilize the structure learning algorithm to supplement the causal relationships not included in the initial network. We use a score-based algorithm with a hill-climbing (HC) search algorithm to complete structure learning. Score-based algorithms are simply applications of various general purpose heuristic search algorithms. They assigns a score to each candidate Bayesian network and try to maximize it with a heuristic search algorithm, such as hill-climbing [40]. To combine the expert knowledge during structure learning, we add the structure constraints to specify where arcs may or may not be included [41]. For example, given a pair of variables *X* and *Y*, if there is no relation between *X* and *Y* which is determined by expert knowledge, then neither X→Y nor Y→X will be added to the final network by the structure learning algorithm with constraints.

Step 4. Network construction. First, we use expectation–maximization to fill the missing values in the dataset. The expectation–maximization algorithm is one of the most effective algorithms for parameter estimation when incomplete data exists. The algorithm alternates iteratively between two steps until it reaches the specified stopping criterion, such as different values of two iterations converging to a certain threshold. Second, the initial BN is determined by expert knowledge based on the Dempster–Shafer theory. Third, the structure learning algorithm is implemented based on the initial network. At last, a directed acyclic graph is constructed, which is the qualitative part of a BN. Then the conditional probability table for each factor is calculated by maximum likelihood estimation, which is the quantitative part of a BN.

### 3.3. Factor Selection Based on Mutual Information

The objective of factor selection is to measure the importance of factors influencing the target and to select the important factors. Different state combinations of factors lead to different learning performance results. We choose the combinations leading to poor performance and then designed appropriate interventions for those learners with specific states of background and learning behavior factors. Generally, several factors exist and each has several states. There will be too many situations if all states are combined. For example, if there are ten factors and each factor has two states, there will be 1024 ($2^{10}$) combinations, making it difficult for instructors to catch key points. If there are four important factors and each factor has two states, there will be 16 ($2^{4}$) combinations. The number of factors decreased by 60% and the number of combinations is reduced by tens of times. The more the number of factors, the faster the number of combinations grows. Thus, factor selection is essential in intervention design.

One of the most commonly used and effective methods to select important factors is mutual information (MI) based on entropy. MI is a measure of the mutual dependence between two random factors. More specifically, it quantifies the amount of information of one random factor by observing the other random factor.

The MI of two random factors can be represented as follows:

(4) $$I\left(X,Y\right)=\sum_{x\in X}\sum_{y\in Y}p\left(x,y\right)\log\frac{p(x,y)}{p(x)p(y)}$$

where p(x,y) is the joint probability function of the factors *X* and *Y*, and p(x) and p(y) are the marginal probability functions of *X* and *Y*, respectively. The entropy measures the expected uncertainty in a factor that is represented as follows:

(5) $$H\left(X\right)=-\sum_{x\in X}p(x)\log p(x).$$

MI is related to entropies of the factors as follows:

(6) $$\begin{array}{cc} I(X,Y) & =H(X)-H(X|Y)\\ & =H(Y)-H(Y|X)\\ & =H(X)+H(Y)-H(X,Y) \end{array}$$

where I(X,Y) represents the MI between factors *X* and *Y*. H(X) and H(Y) are the entropy of *X* and *Y*. H(X,Y) is the joint entropy of *X* and *Y* H(X|Y) is the conditional entropy of *X* given *Y*, which is a measure of how much uncertainty remains about the factor *X* when we know the value of *Y*. Likewise, H(Y|X) is the conditional entropy of *Y* given *X*. The joint and conditional entropies are represented as follows:

(7) $$H\left(X,Y\right)=-\sum_{x\in X}\sum_{y\in Y}p(x,y)\log(x,y)$$

(8) $$H\left(Y|X\right)=-\sum_{x\in X}\sum_{y\in Y}p(x,y)\log(y|x).$$

In general, many factors exist and each factor has several states. Different state combinations lead to different learning performance results. If there are many factors and we make state combinations for all factors, it will increase the complexity for intervention design. Some of those factors may not have much impact on learning performance or other factors. Thus, factor selection is essential for intervention design. We can focus on the important factors and make state combinations of only important factors.

### 3.4. Intervention Design Based on BN and MI

This section aims to provide personalized interventions for different learners. It has been proven that a wide variety of interventions need to be adapted to accommodate learners’ individual differences, rather than a single intervention strategy, which is not sufficient for all learners [42]. It is essential to combine specific background and learning behavior factors of different learners to design interventions.

#### 3.4.1. Intervention Design

Considering that learning behavior factors have a direct impact on learning performance, we first make state combinations of learning behaviors and identify two state combinations leading to the highest probabilities of high grade and low grade. Next, we make combinations of those two state combinations of learning behaviors with all states of important backgrounds. Then the state combinations of learning behavior and background factors leading to higher probabilities of high grade and low grade can be obtained by inference. In this study, we combine MI and inference to design personalized interventions for different learners.

**Figure 3:**
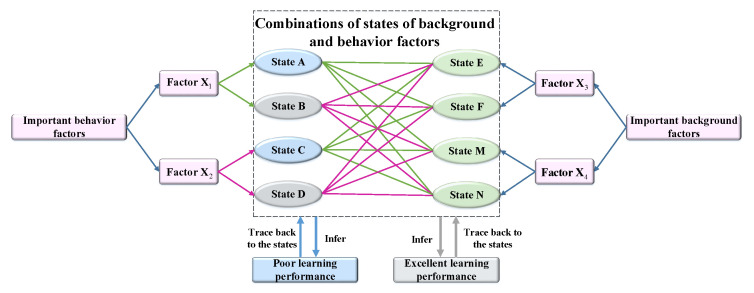
Illustration of personalized intervention design strategy. We make combinations of two state combinations of behavior factors leading to the highest proportion of low grade (A, C) and high grade (B, D) with all states of important background factors ((E, M), (E, N), (F, M), and (F, N)).

The illustration of the personalized intervention design strategy is shown in Figure 3. Based on the results of MI, $X_{1}$ and $X_{2}$ are two important learning behavior factors. $X_{3}$ and $X_{4}$ are two important background factors. Each factor has two states. We first make state combinations of $X_{1}$ and $X_{2}$ and find that the state combination (A, C) leads to the highest probability of a low grade. Similarly, the state combination (B, D) leads to the highest probability of a high grade. Second, we make state combinations of learning behavior factors and background factors (for example, combinations of (A, C) and (E, M), combination (A, C) and (E, N), or combination (B, D) and (F, N), etc.). There are eight total combinations, leading to different learning performances. The group of learners with the highest probability of low grade. represents the poor performance group. Similarly, the group of learners with the highest probability of high grade represents the excellent performance group. Furthermore, we can trace back to the states of factors and draw conclusions about learners with specific backgrounds and behaviors leading to poor or excellent performance. That is important to support making effective educational interventions.

#### 3.4.2. Learning Performance Prediction Using Inference

Once a BN is created, probabilistic inference can be used for learning performance prediction to support intervention design. It is performed using belief updating, which is used to update the probability for a hypothesis when new observations have been received. The objective of inference is to compute the posterior probability $P(Y|X=X^{\prime })$ of query factor *Y*, given a set of observations $X=X^{\prime }$. *X* is a list of observed factors and $X^{\prime }$ is the corresponding list of states (observed values). A factor has several states and *Y* comprises only one query factor. After belief updating, a posterior probability distribution is associated with each factor, reflecting the influence of the set of observations. Inference can be utilized to evaluate the effects of changing of some factors on others, but it does not change the constructed BN.

For example, *X* is a list of new observed factors, such as learning behavior factors ($X_{1},X_{2},X_{3}$), and $X^{\prime }$ is the corresponding list of observed values, such as states of factors ($X_{1}=A,X_{2}=C,X_{3}=E$). The posterior probability of query factor *Y*, such as a low grade level (*L*), can be represented as $P(Y=L|X_{1}=A,X_{2}=C,X_{3}=E)$. The probability of the representation can be inferred using belief updating based on the Bayes theorem [43]. To better design and provide personalized interventions for different learners, we change the states of important factors. The results of MI determine that $X_{1}$ and $X_{2}$ are important factors affecting the query factor *Y*. It means that the state change of $X_{1}$ and $X_{2}$ lead to a larger fluctuation of *Y*, and different state combinations of $X_{1}$ and $X_{2}$ lead to different probabilities of *Y*. If the state combination $\left(X_{1}=B,X_{2}=C,X_{3}=E\right)$ leads to the highest probability of *Y*, that means learners with those specific states have poor learning performance. We can then trace back to analyze those states and apply effective interventions.

## 4. Materials

This study uses the open dataset comprising de-identified data from Canvas Network open courses (running January 2014–September 2015) [44]. We categorized the factors into background factors, behavior factors, and grade to construct the BN. The details of the factors and their states are shown in Table 1 and Table 2.

Depending on the nature of the factor being measured, there are discrete and continuous values. The discrete values, also called states, are mutually exclusive and exhaustive. The continuous values are taken from a given range. It is possible to represent a factor that is naturally represented by continuous values, by using discrete values. To accomplish this, continuous values need to be discretized. In this study, we discretize the continuous values of behavior factors and learning performance into different intervals based on the equal-frequency method [45] and grade level of the Victoria University of Wellington [46], respectively.

For each factor, “Administrative” indicates that the data are generated by users during their interaction with the courses and have been computed by the Canvas Network system. “User-provided” indicates that the data come from questions or surveys of the learner at the time of account registration or at the beginning of the course. We choose data with as much complete background information as possible. For behavior data with empty values, the expectation–maximization algorithm is adopted to fill the empty data. Therefore, there are 1,061 total records. We utilize 80% of the records to construct and training the BN and another 20% of the records for prediction to verify the effectiveness of the BN.

RStudio [47] is an integrated development environment (IDE) for R programming language, which supports extensive R packages. The R package bnlearn can be used for structure learning graphically and contains implementations of various structure learning algorithms and inferences [48]. We use the R package bnlearn to conduct the Bayesian network and make inference for analysis.

## 5. Results

### 5.1. Results of Expert Knowledge

To construct the BN, each invited expert assigns a probability to four relationships for each pair of factors. The Dempster–Shafer theory is then utilized to reduce inconsistencies among experts. The relationship with the highest integrated probability will be chosen as the determined relationship between those two factors. Table 3 shows the probabilities of some relationships and integrated probabilities for those relationships.

Taking the first item as an example, three experts provide probabilities for the relationship of the factor learning type and forum posts. According to (2) and (3), the most probable relationship of “Learning type” and “Forum posts” is obtained and the calculating process is as follows. Then the integrated probabilities for each pair of relationships can be obtained and the final relationship is “Learning type → Forum posts”. Using expert knowledge and the Dempster–Shafer theory, we construct the initial network.

(9) $$\begin{array}{c} K=0.5\times 0.6\times 0.3+0.3\times 0.3\times 0.4+0.2\times 0.1\times 0.3=0.132 \end{array}$$

(10) $$\begin{array}{cc} \; & P(R_{1})=\frac{0.5\times 0.6\times 0.3}{0.132}=0.682\;\;P(R_{2})=\frac{0}{0.132}=0 \end{array}$$

(11) $$\begin{array}{cc} \; & P(R_{3})=\frac{0.3\times 0.3\times 0.4}{0.132}=0.273\;\;P(R_{4})=\frac{0.2\times 0.1\times 0.3}{0.132}=0.045. \end{array}$$

### 5.2. Results of the Constructed BN

**Figure 4:**
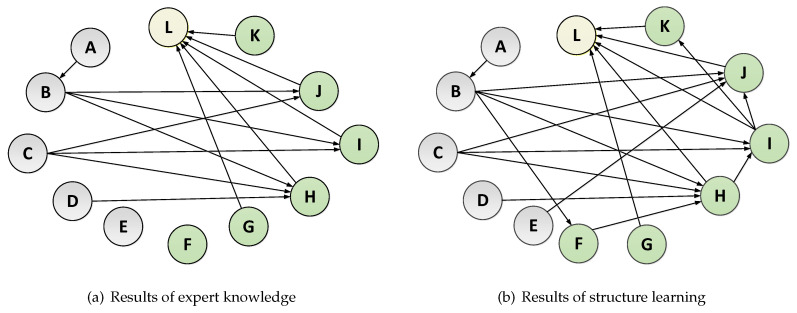
Results of the constructed BN. Subgraph (**a**) shows the initial BN determined by expert knowledge. Subgraph (**b**) shows the final BN after structure learning incorporating expert knowledge.

Figure 4 shows the results of expert knowledge and structure learning respectively and subgraph (b) is the final result. Table 4 shows the factor number and corresponding factor name. In the two graphs, the nodes with the gray color represent the background factors and the nodes with the green color represent the behavior factors. After structure learning, there are six relationships (Views → Completion, Completion → Events, Age → Forum posts, Events → Forum posts, Motivation → Views, and Events → Active days) to be added to the initial network determined by the expert knowledge. The node with the yellow color represents the learning performance. Assignments, completion, forum posts, events, and active days are factors that have a direct influence on grade. Views has a direct impact on completion. Motivation, learning type, expected learning hours, and age are factors that have a direct influence on behavior factors.

When the BN structure is completely directed, we can fit the parameters of the local distributions, which are the quantitative parts, and take the form of the conditional probability tables. From the results, about 40% of learners achieve a level D grade, which represents poor performance on the selected courses and the learners may not master the knowledge prescribed in the syllabus. About 24% of learners achieve a level A grade, which represents excellent performance. The performance of learning behaviors directly affecting the grade is not satisfying. Only a small proportion of learners achieve a high level in completion, forum posts, events, and active days. More than 80% of learners view less than 50% of the content modules. Thus, most learners devote too little on learning and complete a low percent of the total required content modules. Only a few learners participate in their studies continuously. Meanwhile, most of the learners have no intention of communicating through posting on forums. Although most learners complete equal to or more than three assignments, based on their poor performance on other learning behaviors, several learners achieve a level D grade. In the distribution of background factors, more than half of the learners are aged from 19 to 34 years. Several learners study for interest in the topics and for a new career. More than half of the learners deem themselves as active participants. However, there are still about 31.9% of passive learners. About 36% of learners expect to learn two to four hours per week. Learners with a master’s degree or equivalent account for nearly half of the total.

### 5.3. Prediction Results

Learning performance prediction attempts to identify the most likely grade level given a set of observations. We carry out learning performance prediction to verify the effectiveness of the BN. We design three groups of experiments. The first groups use both behavior and background factors (Combined factors) to predict learning performance. The second and third groups use behavior factors and background factors to predict learning performance, respectively. The accuracy is utilized to evaluate the predictive performance. We choose 20% of the data randomly, this was about 212 records as the test data. Logistic regression (LG) and decision tree (DT) are the most commonly used algorithms, which are chosen as the compared methods. The BN is the method used in this paper, and the experimental results are shown in Table 5.

From the results, the Bayesian network based on combined factors (BN-C) performs best, which achieves 82.14% accuracy, about 30.67%, 7.41% higher than logistic regression and decision tree based on combined factors (LG-C, DT-C), respectively. Additionally, the prediction results of methods based on combined factors perform much better than methods based on behavior factors (LG-Be, DT-Be, BN-Be) and background factors (LG-Ba, DT-Ba, BN-Ba). For example, LG-C achieves about 6.63% and 38.61% higher than LG-Be and LG-Ba, respectively, in accuracy. Similarly, BN-C achieves about 2.3% and 45.71% higher than BN-Be and BN-Ba, respectively, in accuracy, confirming the effectiveness of the constructed network with combined factors.

### 5.4. Results of Factor Selection Using MI

Factor selection aims to select the important factors influencing the target factor. We use MI to implement factor selection, and the factor with the maximum value has the highest effect on the grade. The mutual information of factors influencing grade is shown in Table 6.

From the results, we select two important behavior factors and two important background factors. The selected factors have a high MI influencing the target factor in comprehensive and their MI is shown in bold. Completion (MI:0.36489) and forum posts (MI:0.1489) are the two most important behavior factors influencing grade. Learning type (MI:0.06549) and motivation (MI:0.02803) are the two most important background factors influencing grade. As behavior factors have a direct impact on learning performance, the MI of completion and forum posts are much higher than that of learning type and motivation. Moreover, we also select important factors influencing the important behavior factors. According to the results shown in Table 7 and Table 8, learning type and motivation both have important impacts on completion and forum posts.

In conclusion, we choose completion and forum posts as important behavior factors, and learning type and motivation as important background factors. Further analysis is performed based on the results of this factor selection. We will explore learners with different performances considering the most important factors, aiming to design personalized interventions strategies for them.

#### 5.4.1. Impact of Behavior Factors

To explore the impact of important behavior factors on learning performance, we make state combinations of completion and forum posts, and infer the grade level. There are nine state combinations, and the probabilities of grade levels (Grade = Level A, B, C, or D) for each combination are shown in Table 9.

From the results, learners with a low level of completion and forum posts are prone to achieve grade level D (87.5%) and learners with a high level of completion of courses and forum posts are prone to achieve grade level A (74%). Further analysis will be conducted combining those two state combinations with state combinations of important background factors leading to poor and excellent performance.

#### 5.4.2. Impact of Background Factors

To explore the impact of important background factors on learning performance, we make state combinations of motivation and learning type under the condition of “Completion = High” and “Forum posts = High”, and infer the grade level. The top three highest probabilities of grade level D and level A for state combinations of important background factors are shown in Table 10 and Table 11, respectively. From the results, passive learners with the motivation of preparing for college are prone to achieve grade level D (79.9%) and active learners with the motivation of gaining skills to use at work or for a promotion are prone to achieve grade level A (44.3%).

#### 5.4.3. Impact of the Combinations of Behavior and Background Factors

To design interventions, the groups of learners with specific states leading to poor performance should be inferred. For comparison, we also infer the groups of learners leading to excellent performance. According to the personalized intervention design method, due to the more remarkable impact of behavior factors on learning performance, we first fix the state combination of completion and forum posts, leading to much higher probability of level D grade (“Completion = Low” and “Forum posts = Low”) or level A grade (“Completion = High” and “Forum posts = High”). Next, we make all state combinations of motivation and learning type with the fixed state combinations of completion and forum posts. Thus, we can infer that what behavior and background states lead to much better or worse learning performance. We will then identify learners in need of help and design personalized interventions for different groups of learners. Additionally, computation is performed fewer times than when making all state combinations of all behavior and background factors. The top three highest proportions of grade level D and grade level A for the state combinations of important background and behavior factors are shown in Table 12 and Table 13, respectively.

From the results, compared with single factors, much worse and better performance can be obtained combining background and behavior factors. For example, drop-in learners with a low level of completion and forum posts, but who enjoy being part of a community of learners, have a 95.2% probability to achieve a level D grade and only a 2.68% probability to achieve a level A grade. Similarly, active learners with a high level of completion and forum posts, and learning for school have a 77% probability to achieve a level A grade and have a 12.6% probability to achieve a level D grade. Thus, we can identify the groups of learners who may need help. Furthermore, we can design personalized interventions for learners considering their background factors.

### 5.5. Personalized Interventions

#### 5.5.1. Interventions for Different Learners

The important application of MI and learning performance prediction is to anticipate effective intervention strategies based on more than one contributory factor, aiming to improve learning performance. Specifically, we identify learners with poor performance and design interventions considering their background and learning behaviors. In this study, motivation, learning type, completion, and forum posts are important background and learning behavior factors influencing learning performance. Thus, we make interventions considering the state combinations of those factors. For example, Table 12 shows three situations of poor learning performance with different state combinations. Considering the learning motivation (e.g., community and topics), we can make some interventions related to enhancing social interactions and interesting topics. Considering the learning type (e.g., Drop-in), we can make some interventions related to reward mechanism and game-based learning to encourage learning. Likewise, considering the learning behaviors (“Completion = Low” and “Forum posts = Low”), we can make some interventions related to enhancing social interactions, reward mechanisms, and game-based learning. For comparison, we also identify learners with excellent performance similarly. From the two types of learners, we may obtain a deep and comprehensive understanding of the discrepancy in learning outcomes.

However, not all learners will be provided with interventions. For example, a positive correlation between effort and learning performance can be easily obtained [49]. This conclusion has little practical significance for intervention design besides encouraging learners to work harder in learning. In this situation, we are not sure what factors cause less investment in learning and whether interventions should be made for all poor performance learners. Learners enroll in courses for various reasons. Satisfying curiosity and advancing in a current job are common motivating factors [50,51]. Many learners join online courses only to have some exposure to the best platforms in the world [52]. Learners motivated for a work promotion may result in more investment than for curiosity, leading to better learning performance. There is no urgent need to design interventions for learners motivated by curiosity.

According to previous studies, five categories of interventions in an online learning environment are summarized as follows: (1) observation, (2) knowledge-building interventions, (3) interactive interventions, (4) curriculum and pedagogical interventions, and (5) text-based warning interventions. Different from previous studies, an important conclusion in this paper is that we do not have to design interventions for all poor performance learners. For a learner who lacks motivation, the best intervention is no intervention and tracking observation. If the learner continues engagement in learning and performs worse, we will design corresponding interventions.

Knowledge-building interventions develop new understandings and thinking to improve learning and generate further knowledge [53]. Educational resource recommendations are an effective strategy to optimize learning and broaden knowledge [54]. If a learner has difficulty with understanding the current lecture, we can recommend some related educational resources, which may explain theories in an easily understandable way and have sufficient examples. To better complete personalized educational resources, which are matched to learners’ need, we should take some measures to estimate learners’ knowledge level, such as a knowledge assessment [55]. In this way, we can identify the knowledge that is only weakly mastered by learners and improve their learning.

The goal of interactive interventions is either to promote learner–learner communication [56] or to support learner–instructor feedback [57], such as collaborative learning [58], forum discussion [59], game-based activities [60], and post-lecture exercises [61]. Communication can promote learning enthusiasm and make learners invest more in learning. Curriculum and pedagogical interventions are used to help learners engage in learning and generate interest for courses, such as sending learning materials and automatic reminders [62], adding interactive elements in the lecture [63], post-hoc analysis (e.g., click data analysis) [64] and reward mechanisms [65]. Text-based warning interventions are designed for psychological considerations, including identification of negative or anxious sentiments [66] and topic modeling of forum posts [67]. Sentiment analysis and topic modeling of those valuable opinionated texts can assist instructors to make guiding instructions to improve learning performances. The detailed conclusion of the proposed personalized interventions are shown in Table 14.

#### 5.5.2. Case Study

This section demonstrates a case of poor performance with the state combination of specific background and learning behavior factors. In Table 12, drop-in learners who are interested in participating in the community (“Motivation = Community” and “Learning type = Drop-in”) leads to the highest probability of a level D grade of the combination of motivation and learning type with the learning behaviors of “Completion = Low” and “Forum posts = Low”. In this case, learners have some difficulties in continuous learning and are prone to drop courses in the online environment. Learning willingness and behaviors of those learners may change rapidly over the span of a course. At the beginning, those learners probably have great enthusiasm to watch videos and participate discussions. With the course in session, the learners may become inactive or dropout from courses. Therefore, it is extremely necessary for instructors to make some guiding suggestions or interventions to improve learning for those learners. Table 15 demonstrates the personalized interventions for the given case.

## 6. Discussion

The experimental results have proven the effectiveness of the proposed framework. The constructed BN not only demonstrates the causal relationships between factors and learning performance visually but also measures those relationships quantitatively. The prior probabilities of the BN demonstrate that several learners do not perform well on the selected courses; about 40% of learners achieve a level D grade. A previous study has shown an even higher proportion of a low grade [3]. It is essential to design some interventions to improve learning performance. The results of factor selection show that completion, forum posts, learning type, and motivation are important factors. Moreover, the results of learning performance prediction verify the effectiveness of the constructed model, and combining backgrounds and learning behaviors is the best way to identify learners in need of help. Finally, the personalized interventions are given for different learners with poor performance. In practice, there will be more cases with different state combinations of different factors. Naturally, there is room for further work and improvements. We discuss a few points here.

**Criteria of learning performance.** According to the learning performance criteria of Victoria University of Wellington, a grade less than 50 is discretized to grade D, which represents poor performance. The proportion of poor performance may be different based on different criteria. In future work, the criteria from other research institutes and various criteria, such as added knowledge and skill building, will be considered [10].

**Other factors.** Many other factors are not researched in this paper, which have not been proven to have an important impact on learning performance, such as gender, total scores from previous education [68], cumulative time spent on learning, and the number of viewed posts [69]. Our future work is to analyze and model those factors alongside the factors used in this paper, which may help to gain a deeper insight into why learners achieve poor learning performances, and how to improve learning performance.

**Other methods.** Some other machine learning methods can be applied in causal analysis. The authors in [26] improved the Apriori algorithm to make causal analysis between learning behaviors and performance. This method is based on association analysis that can not express the connection between different rules. BN can graphically represent the joint probability distribution among factors and comprehensively considers the effect of several factors on target factors. The structure equation model is also a graphical model that is able to model causal relationships between factors. The method is applied in education [27] and other fields. The structure equation model heavily relies on expert knowledge and uses data to justify the expert knowledge. BN can combine the expert knowledge with a constructed network that gives the maximum likelihood based on data. In future work, we will preform a causal analysis using other methods and make a comparison.

**Other applications.** The framework proposed in this paper can be applied not only to causal relationship modeling between factors and learning performance but also to other educational research fields, which have similar needs for causal relationship modeling and analysis to propose some guiding suggestions.

## 7. Conclusions

The goal of this paper is to construct a well-defined Bayesian network and then provide personalized interventions for different learners to improve learning. To construct a reasonable network, we combine expert knowledge based on the Dempster–Shafer theory, which exploits prior knowledge and structure learning, taking advantage of the data. To make accurate predictions, we combine background and behavior factors, which perform much better than the single-factor method. To design effective interventions, we choose the important factors, which help instructors focus on the most relevant points. Based on the state combinations of important factors, we identify the learners in need of the most help, not simply all poor performance learners. And last, we conclude several interventions for different learners which may support making effective decisions and successful applications in education. In future work, we will continue our research considering more factors affecting learning performance, criteria of learning performances, and methods for modeling the causal relationships between factors.

## Figures and Tables

**Table 1:** Detailed description of background factors and their states in the dataset.

Factor Name	Factor Description	State Name	State Description
Education(User-provided)	Highest level of education completed	High school	High school or college preparatory school
		Completed two-year college	Completed two-year college degree
		Completed four-year college	Completed four-year college degree
		Master’s degree	Master’s degree or equivalent
		Uncertain	None of these
Age (User-provided)	Age brackets	19 to 34	From 19 to 34 years old
		35 to 54	From 35 to 54 years old
		55 or older	Older than 54 years old
Motivation (User-provided)	Standardized reason for taking course (from survey)	Curiosity	Is curious about online courses and likes the online format
		Topics	Enjoys learning about topics that interest me
		A new career	Hopes to gain skills for a new career
		Work	Hopes to gain skills to use at work or for a promotion
		College	Is preparing for college for the first time
		School	Is preparing to go back to school
		Community	Enjoys being part of a community of learners
Learning type (User-provided)	Standardized learning type (from survey)	Passive	Passive participant
		Active	Active participant
		Drop-in	Drop-in participant
Expected learning hours (User-provided)	Standardized range of hours per week (from survey)	Less than 1	Less than 1 hour per week
		1 to 2	Between 1 and 2 h per week
		3 to 4	Between 2 and 4 h per week
		5 to 6	Between 4 and 6 h per week
		7 to 8	Between 6 and 8 h per week
		More than 8	More than 8 h per week

**Table 2:** Detailed description of behavior factors and their states in the dataset.

Factor Name	Factor Description	State Name	State Description
Views (Administrative)	Whether views are equal to or more than 50% of the content modules	Yes	Equal to or more than 50% of the content modules
		No	Less than 50% of the content modules
Assignments (Administrative)	Complete equal to or more than three assignments	Yes	Equal to or more than three assignments
		No	Less than three assignments
Completion (Administrative)	Percent of content modules a learner has completed	Low	Low completion rate (0 <= Completion <= 0.08)
		Moderate	Moderate completion rate (0.09 <= Completion <= 0.17)
		High	High completion rate (0.18 <= Completion <= 0.92)
Forum posts (Administrative)	Number of posts total in discussion forums throughout the course	Low	Low number of forum posts (0 <= Forum posts <= 1)
		Moderate	Moderate number of forum posts (2 <= Forum posts <= 4)
		High	High number of forum posts (5 <= Forum posts <= 64)
Events (Administrative)	Number of distinct interaction events with the course, such as page views	Low	Low number of events (0 <= Events <= 110))
		Moderate	Moderate number of events (111 <= Events <= 280)
		High	High number of events (281 <= Events <= 1002)
Active days (Administrative)	Number of active days with one or more events	Low	Low number of active days (1 <= Active days <= 4)
		Moderate	Moderate number of active days (5 <= Active days <= 8)
		High	High number of active days (9 <= Active day <= 78)
Grade (Administrative)	Final grade in the course	Level A	Excellent performance (0.8 <= Grade <= 1)
		Level B	Moderate performance (0.65 <= Grade <= 0.79)
		Level C	Moderate performance (0.5 <= Grade <= 0.64)
		Level D	Poor performance (0 <= Grade <= 0.49)

**Table 3:** The integrated probabilities for each pair of relationships between factors based on the opinions elicited from three experts.

Expert	Learning Type → Forum Posts	Learning Type ← Forum Posts	Learning Type | Forum Posts	Learning Type ? Forum Posts
Expert 1	$P_{1}\left(R_{1}\right)=0.5$	$P_{1}\left(R_{2}\right)=0$	$P_{1}\left(R_{3}\right)=0.3$	$P_{1}\left(R_{4}\right)=0.2$
Expert 2	$P_{2}\left(R_{1}\right)=0.6$	$P_{2}\left(R_{2}\right)=0$	$P_{2}\left(R_{3}\right)=0.3$	$P_{2}\left(R_{4}\right)=0.1$
Expert 3	$P_{3}\left(R_{1}\right)=0.3$	$P_{3}\left(R_{2}\right)=0$	$P_{3}\left(R_{3}\right)=0.4$	$P_{3}\left(R_{4}\right)=0.3$
**Integrated probability**	$P(R_{1})=0.682$	$P(R_{2})=0$	$P(R_{3})=0.273$	$P(R_{4})=0.045$
Expert	Grade → Completion	Grade ← Completion	Grade | Completion	Grade ? Completion
Expert 1	$P_{1}\left(R_{1}\right)=0.2$	$P_{1}\left(R_{2}\right)=0.5$	$P_{1}\left(R_{3}\right)=0.1$	$P_{1}\left(R_{4}\right)=0.2$
Expert 2	$P_{2}\left(R_{1}\right)=0.2$	$P_{2}\left(R_{2}\right)=0.6$	$P_{2}\left(R_{3}\right)=0$	$P_{2}\left(R_{4}\right)=0.2$
Expert 3	$P_{3}\left(R_{1}\right)=0.1$	$P_{3}\left(R_{2}\right)=0.5$	$P_{3}\left(R_{3}\right)=0$	$P_{3}\left(R_{4}\right)=0.3$
**Integrated probability**	$P(R_{1})=0.02$	$P(R_{2})=0.918$	$P(R_{3})=0$	$P(R_{4})=0.061$
Expert	Forum posts → Active days	Forum posts ← Active days	Forum posts | Active days	Forum posts ? Active days
Expert 1	$P_{1}\left(R_{1}\right)=0.3$	$P_{1}\left(R_{2}\right)=0.2$	$P_{1}\left(R_{3}\right)=0.4$	$P_{1}\left(R_{4}\right)=0.1$
Expert 2	$P_{2}\left(R_{1}\right)=0.2$	$P_{2}\left(R_{2}\right)=0.4$	$P_{2}\left(R_{3}\right)=0.4$	$P_{2}\left(R_{4}\right)=0$
Expert 3	$P_{3}\left(R_{1}\right)=0.4$	$P_{3}\left(R_{2}\right)=0.1$	$P_{3}\left(R_{3}\right)=0.5$	$P_{3}\left(R_{4}\right)=0$
**Integrated probability**	$P(R_{1})=0.214$	$P(R_{2})=0.071$	$P(R_{3})=0.714$	$P(R_{4})=0$
Expert	Completion → Events	Completion ← Events	Completion | Events	Completion ? Events
Expert 1	$P_{1}\left(R_{1}\right)=0$	$P_{1}\left(R_{2}\right)=0.3$	$P_{1}\left(R_{3}\right)=0$	$P_{1}\left(R_{4}\right)=0.7$
Expert 2	$P_{2}\left(R_{1}\right)=0.1$	$P_{2}\left(R_{2}\right)=0.3$	$P_{2}\left(R_{3}\right)=0.$	$P_{2}\left(R_{4}\right)=0.6$
Expert 3	$P_{3}\left(R_{1}\right)=0.2$	$P_{3}\left(R_{2}\right)=0.3$	$P_{3}\left(R_{3}\right)=0$	$P_{3}\left(R_{4}\right)=0.5$
**Integrated probability**	$P(R_{1})=0$	$P(R_{2})=0.114$	$P(R_{3})=0$	$P(R_{4})=0.886$

**Table 4:** Factor representation and the corresponding factor name.

Representation	Factor Name	Representation	Factor Name
A	Education	G	Assignments
B	Motivation	H	Completion
C	Learning type	I	Events
D	Expected learning hours	J	Forum posts
E	Age	K	Active days
F	Views	L	Grade

**Table 5:** Prediction results in accuracy (%). There are three groups of experiments—methods using combined factors (LG-C, DT-C, BN-C), behavior factors (LG-Be, DT-Be, BN-Be), and background factors (LG-Ba, DT-Ba, BN-Ba). “C” is the abbreviation for “combined factors”. “Be” is the abbreviation for “behavior factors” and “Ba” is the abbreviation for “background factors”. LG-C represents logistic regression using combined factors. The representation of other methods is similar. From the results, methods using combined factors perform much better, from which, our proposed method BN-C performs best.

Combined Factors		Behavior Factors		Background Factors	
Method	Accuracy	Method	Accuracy	Method	Accuracy
LG-C	62.86	LG-Be	58.95	LG-Ba	45.35
DT-C	76.47	DT-Be	65.38	DT-Ba	54.24
**BN-C**	**82.14**	BN-Be	80.29	BN-Ba	56.37

**Table 6:** MI of factors influencing grade.

Factor	MI
Completion	**0.36489**
Forum posts	**0.1489**
Active days	0.11021
Events	0.10308
Views	0.02308
Assignments	0.0017
Learning type	**0.06549**
Motivation	**0.02803**
Age	0.0073
Education	0.0065
Expected Learning Hours	0.00367

**Table 7:** MI of factors influencing completion.

Factor	MI
Motivation	**0.09092**
Learning Type	**0.07323**
Views	0.06093
Expected Learning Hours	0.01278

**Table 8:** MI of factors influencing forum posts.

Factor	MI
Learning Type	**0.08779**
Events	0.03974
Age	0.03879
Motivation	**0.01969**

**Table 9:** State combinations of completion and forum posts, and their probabilities of grade level (%). About 74% learners achieve grade level A with “Completion = High” and “Forum posts = High” and about 87.5% learners achieve grade level D with “Completion = Low” and “Forum posts = Low”.

Completion	Forum Posts	Grade = Level A	Grade = Level B	Grade = Level C	Grade = Level D
**High**	**High**	**74**	9.48	6.97	9.58
High	Moderate	51.1	6.62	27.7	14.6
High	Low	37.5	8.4	19.4	34.7
Moderate	High	66.3	2.12	25.7	5.87
Moderate	Moderate	12.8	0.11	71.5	15.5
Moderate	Low	9.08	0.12	63.8	27
Low	High	48.7	16.5	2.09	32.7
Low	Moderate	7.27	3.22	2.78	86.7
**Low**	**Low**	2.09	2.35	8.09	**87.5**

**Table 10:** Probabilities of each grade level of state combinations of motivation and learning type (Top three items ordered by Grade = “Level D”) (%). About 79.9% learners achieve grade level D with “Motivation = College” and “Learning type = Passive”.

Motivation	Learning Type	Level A	Level B	Level C	Level D
**College**	**Passive**	6.12	5.2	8.82	**79.9**
School	Passive	7.67	1.27	13.2	77.9
College	Drop-in	13.6	3.14	8.15	75.1

**Table 11:** Probabilities of each grade level of state combinations of motivation and learning type (Top three items ordered by Grade = “Level A”) (%). About 44.3% learners achieve grade level A with “Motivation = Work” and “Learning type = Active”.

Motivation	Learning Type	Level A	Level B	Level C	Level D
**Work**	**Active**	**44.3**	9.53	17.8	28.3
Topics	Active	35.1	4.74	27.3	32.8
School	Active	32.8	9.04	9.7	48.5

**Table 12:** Probabilities of each grade level of state combinations of motivation, learning type, and fixed behavior states (Top three items ordered by Grade = “Level D”) (%). About 95.2% learners achieve grade level D with “Motivation = Community”, “Learning type = Drop-in”, “Completion = Low” and “Forum posts = Low”.

Motivation	Learning Type	Completion	Forum Posts	Level A	Level B	Level C	Level D
**Community**	**Drop-in**	**Low**	**Low**	2.68	2.01	0.12	**95.2**
Topics	Drop-in	Low	Low	1.92	1.35	4.14	92.6
Curiosity	Active	Low	Low	2.73	2.84	5.09	89.3

**Table 13:** Probabilities of each grade level of state combinations of motivation, learning type, and fixed behavior states (Top three items ordered by Grade = “Level A”) (%). About 77% learners achieve grade level A with “Motivation = School”, “Learning type = Active”, “Completion = High” and “Forum posts = High”.

Motivation	Learning Type	Completion	Forum Posts	Level A	Level B	Level C	Level D
**School**	**Active**	**High**	**High**	**77**	10.2	0.21	12.6
Work	Drop-in	High	High	76.3	10.3	1.12	12.2
Work	Active	High	High	75.4	9.68	4.1	10.8

**Table 14:** Detailed description of the five types of personalized interventions.

Category	Item	Description
Observation	Observation	Take no intervention and keep tracing learners’ learning.
Knowledge-building interventions	Knowledge mastery level assessment	Model learners’ mastery level of given knowledge conveyed by lectures. Ifonly a small proportion of learners do not master the knowledge, we suggest designing interventions, such as educational resources recommendations, toimprove learners’ learning. Ifmost learners do not master the knowledge, we suggest instructors to improve teaching and lecture quality.
	Exercise recommendation	Recommend exercises related to knowledge that learners have not mastered well, according to the learners’ knowledge mastery level.
	Book recommendation	Recommend books according to the learners’ knowledge mastery level and education backgrounds. These books may explain theories in an easily understandable way and have sufficient examples.
	Video recommendation	Recommend high quality videos related to the selected course according to the learners’ knowledge mastery level and education backgrounds.
Interactive interventions	Collaborative learning	Divide learners into small collaborative learning groups. Each learning group comprises learners with different knowledge mastery levels and the same backgrounds, toenhance and improve their learning.
	Discussion forum	Guide learners to participate in the discussion forum to ask questions or help others. Setting up different topics related to not well-mastered knowledge or extended knowledge outside lectures for discussion.
	Game-based activities	Organize game-based activities between learning groups, such as virtual reality-based teaching, andquestion-and-answer contests between groups.
	Post-lecture exercises	Design exercises after lectures to assess learners’ knowledge mastery levels. Set several knowledge points for each exercise and perform statistics of the answering time, times of asking for help, andso on.
Curriculum and pedagogical interventions	Automatic reminder	Send learning materials before the lecture and learning progress to learners automatically.
	Add interactive elements in the lecture	Add interactive exercises and questions during the video to stimulate thinking.
	Post-hoc analysis	Perform post-hoc analysis of clickstream data and major video interaction events, toenhance learner engagement by improving the quality and interactivity.
	Reward mechanism	Give incentives for changing behaviors, such as accumulated points and vouchers, which may be a convertible opportunity to have priority of communication with instructors.
Text-based warning interventions	Identification of negative or anxious sentiment	Identify forum posts with negative or anxious sentiment. Those posts will be used for topic modeling to improve teaching or learning.
	Topic modeling of forum posts	Conduct topic modeling of the forum posts. Ifmost learners have negative or anxious comments, we suggest improving the teaching or lecture quality with the results of the topic modeling, such as “poor sound quality”, “too obscure to understand”, “speaks too fast”, etc. Ifa small proportion of learners have negative or anxious comments, we suggest designing interventions for the results of topic modelling, such as “hard to understand the ’stack’ concept”, “need more detailed explanation”, andso on.

**Table 15:** Personalized interventions for learners of the given case.

Background	Learning Behavior	Intervention
Motivation = Community Learning type = Drop-in	Completion = LowForum posts = Low	(1) Collaborative learning. Guiding learners to join some collaborative learning groups with the same age, education and knowledge mastery level or organizing new groups of topics related to the participated courses. (2) Reward mechanism. As the learners’ learning type is drop-in, adopt some reward interventions such as accumulated points for continued learning and posting forums, which may encourage their learning. (3) Automatic reminders and educational resource recommendations. Sending some simple and interesting books and videos before the lecture to stimulate interest in learning. (4) Game-based activities. Organizing game-based activities in or between collaborative learning groups. (5) Identification of negative or anxious sentiments. Identify learners with negative or anxious sentiments. (6) Topic modeling of forum posts. We can analyze the forum posts by text mining to identify why the learners drop lectures.

## References

[1] Al-Shabandar R., Hussain A.J., Liatsis P., Keight R. (2018). Analyzing Learners Behavior in MOOCs: An Examination of Performance and Motivation Using a Data-Driven Approach. IEEE Access.

[2] Alraimi K.M., Zo H., Ciganek A.P. (2015). Understanding the MOOCs continuance: The role of openness and reputation. Comput. Educ..

[3] Anderson A., Huttenlocher D.P., Kleinberg J.M., Leskovec J. Engaging with massive online courses. Proceedings of the 23rd International World Wide Web Conference (WWW’14).

[4] Hsin W.J., Cigas J. (2013). Short videos improve student learning in online education. J. Comput. Sci. Coll..

[5] Szpunar K.K., Khan N.Y., Schacter D.L. (2013). Interpolated memory tests reduce mind wandering and improve learning of online lectures. Proc. Natl. Acad. Sci. USA.

[6] Hwang G.J., Wu P.H., Chen C.C. (2012). An online game approach for improving students’ learning performance in web-based problem-solving activities. Comput. Educ..

[7] Abu Tair M.M., El-Halees A.M. (2012). Mining educational data to improve students’ performance: A case study. Int. J. Inf. Commun. Technol. Res..

[8] Kumar S., Bharadwaj B., Pal S. (2011). Data Mining Applications: A Comparative Study for Predicting Students Performance. Int. J. Innov. Technol. Creat. Eng..

[9] Xing W., Guo R., Petakovic E., Goggins S. (2015). Participation-based student final performance prediction model through interpretable Genetic Programming: Integrating learning analytics, educational data mining and theory. Comput. Hum. Behav..

[10] Picciano A.G. (2002). Beyond student perceptions: Issues of interaction, presence, and performance in an online course. J. Asynchronous Learn. Netw..

[11] Meier Y., Xu J., Atan O., Van der Schaar M. (2016). Predicting grades. IEEE Trans. Signal Process..

[12] Elbadrawy A., Polyzou A., Ren Z., Sweeney M., Karypis G., Rangwala H. (2016). Predicting student performance using personalized analytics. Computer.

[13] Daradoumis T., Bassi R., Xhafa F., Caballé S. A review on massive e-learning (MOOC) design, delivery and assessment. Proceedings of the 2013 Eighth International Conference on P2P, Parallel, Grid, Cloud and Internet Computing.

[14] Coffrin C., Corrin L., de Barba P., Kennedy G. Visualizing patterns of student engagement and performance in MOOCs. Proceedings of the Fourth International Conference on Learning Analytics And Knowledge.

[15] Conijn R., Van den Beemt A., Cuijpers P. (2018). Predicting student performance in a blended MOOC. J. Comput. Assist. Learn..

[16] Phan T., McNeil S.G., Robin B.R. (2016). Students’ patterns of engagement and course performance in a Massive Open Online Course. Comput. Educ..

[17] Al-Shabandar R., Hussain A., Laws A., Keight R., Lunn J., Radi N. Machine learning approaches to predict learning outcomes in Massive open online courses. Proceedings of the 2017 International Joint Conference on Neural Networks (IJCNN).

[18] Shapiro H.B., Lee C.H., Roth N.E.W., Li K., Çetinkaya-Rundel M., Canelas D.A. (2017). Understanding the massive open online course (MOOC) student experience: An examination of attitudes, motivations, and barriers. Comput. Educ..

[19] Goodwin L.D., Leech N.L. (2006). Understanding Correlation: Factors That Affect the Size of r. J. Exp. Educ..

[20] Asuero A.G., Sayago A., Gonzalez A. (2006). The correlation coefficient: An overview. Crit. Rev. Anal. Chem..

[21] Pearl J. (2002). Causality: Models, reasoning, and inference. IIE Trans..

[22] Mohammadfam I., Ghasemi F., Kalatpour O., Moghimbeigi A. (2017). Constructing a Bayesian network model for improving safety behavior of employees at workplaces. Appl. Ergon..

[23] Parhizkar T., Balali S., Mosleh A. (2018). An entropy based bayesian network framework for system health monitoring. Entropy.

[24] Aguilera P., Fernández A., Fernández R., Rumí R., Salmerón A. (2011). Bayesian networks in environmental modelling. Environ. Model. Softw..

[25] Reich J. (2015). Rebooting MOOC research. Science.

[26] Wang H., Hao X., Jiao W., Jia X. Causal Association Analysis Algorithm for MOOC Learning Behavior and Learning Effect. Proceedings of the 2016 IEEE 14th Intl Conf on Dependable, Autonomic and Secure Computing, 14th Intl Conf on Pervasive Intelligence and Computing, 2nd Intl Conf on Big Data Intelligence and Computing and Cyber Science and Technology Congress (DASC/PiCom/DataCom/CyberSciTech).

[27] Ramirez-Arellano A., Acosta-Gonzaga E., Bory-Reyes J., Hernández-Simón L.M. (2018). Factors affecting student learning performance: A causal model in higher blended education. J. Comput. Assist. Learn..

[28] Kendall J. (2003). Designing a research project: Randomised controlled trials and their principles. Emerg. Med. J. EMJ.

[29] Steiner P.M., Wroblewski A., Cook T.D. (2009). Randomized experiments and quasi-experimental designs in educational research. The SAGE International Handbook of Educational Evaluation.

[30] Cook T.D., Shadish W.R., Wong V.C. (2008). Three conditions under which experiments and observational studies produce comparable causal estimates: New findings from within-study comparisons. J. Policy Anal. Manag. J. Assoc. Public Policy Anal. Manag..

[31] Ren J., Jenkinson I., Wang J., Xu D., Yang J. (2008). A methodology to model causal relationships on offshore safety assessment focusing on human and organizational factors. J. Saf. Res..

[32] Torgerson C.J., Torgerson D.J. (2001). The need for randomised controlled trials in educational research. Br. J. Educ. Stud..

[33] Bradshaw C.P., Mitchell M.M., Leaf P.J. (2010). Examining the effects of schoolwide positive behavioral interventions and supports on student outcomes: Results from a randomized controlled effectiveness trial in elementary schools. J. Posit. Behav. Interv..

[34] Connolly P., Keenan C., Urbanska K. (2018). The trials of evidence-based practice in education: A systematic review of randomised controlled trials in education research 1980–2016. Educ. Res..

[35] Millán E., DescalçO L., Castillo G., Oliveira P., Diogo S. (2013). Using Bayesian networks to improve knowledge assessment. Comput. Educ..

[36] Millán E., Jiménez G., Belmonte M.V., Pérez-de-la Cruz J.L. (2015). Learning Bayesian networks for student modeling. Proceedings of the International Conference on Artificial Intelligence in Education.

[37] De Campos C.P., Zeng Z., Ji Q. Structure learning of Bayesian networks using constraints. Proceedings of the 26th Annual International Conference on Machine Learning.

[38] Niculescu R.S., Mitchell T.M., Rao R.B. (2006). Bayesian network learning with parameter constraints. J. Mach. Learn. Res..

[39] Perrier E., Imoto S., Miyano S. (2008). Finding optimal Bayesian network given a super-structure. J. Mach. Learn. Res..

[40] Scutari M. (2009). Learning Bayesian networks with the bnlearn R package. arXiv.

[41] de Campos L.M., Castellano F.J.G. (2007). Bayesian network learning algorithms using structural restrictions. Int. J. Approx. Reason..

[42] Felder R. (1993). Reaching the Second Tier: Learning and Teaching Styles in College Science Education. J. Coll. Sci. Teach..

[43] Cowell R. (1998). Introduction to inference for Bayesian networks. Learning in Graphical Models.

[44] Dataverse Canvas Network Person-Course (1/2014-9/2015) De-Identified Dataset [DB/OL]. (2016-02-16). https://dataverse.harvard.edu/dataset.xhtml?persistentId=doi:10.7910/DVN/1XORAL.

[45] Liu H., Hussain F., Tan C., Dash M. (2002). Discretization: An enabling technique. Data Min. Knowl. Discov..

[46] Victoria University of Wellington (2014). Standard Pass/Fail Grades [DB/OL]. https://www.victoria.ac.nz/students/study/progress/grades.

[47] Allaire J. (2012). RStudio: Integrated Development Environment for R.

[48] Kalisch M., Mächler M., Colombo D., Maathuis M.H., Bühlmann P. (2012). Causal inference using graphical models with the R package pcalg. J. Stat. Softw..

[49] Kolari S., Savander-Ranne C., Viskari E.-L. (2008). Learning needs time and effort: A time-use study of engineering students. Eur. J. Eng. Educ..

[50] Breslow L., Pritchard D.E., DeBoer J., Stump G.S., Ho A.D., Seaton D.T. (2013). Studying learning in the worldwide classroom research into edX’s first MOOC. Res. Pract. Assess..

[51] Christensen G., Steinmetz A., Alcorn B., Bennett A., Woods D., Emanuel E. (2013). The MOOC Phenomenon: Who Takes Massive Open Online Courses and Why?. Available SSRN 2350964.

[52] Rai L., Chunrao D. (2016). Influencing factors of success and failure in MOOC and general analysis of learner behavior. Int. J. Inf. Educ. Technol..

[53] Bell D. (2014). The Perceived Success of Interventions in Science Education-a Summary: A Report for the Wellcome Trust.

[54] Okoye I., Maull K., Foster J., Sumner T. (2012). Educational recommendation in an informal intentional learning system. Educational Recommender Systems and Technologies: Practices and Challenges.

[55] Leighton J., Gierl M. (2007). Cognitive Diagnostic Assessment for Education: Theory and Applications.

[56] Lee Y.C., Lin W.C., Cherng F.Y., Wang H.C., Sung C.Y., King J.T. Using time-anchored peer comments to enhance social interaction in online educational videos. Proceedings of the 33rd Annual ACM Conference on Human Factors in Computing Systems.

[57] Glassman E.L., Kim J., Monroy-Hernández A., Morris M.R. Mudslide: A spatially anchored census of student confusion for online lecture videos. Proceedings of the 33rd Annual ACM Conference on Human Factors in Computing Systems.

[58] Garcia R.A., Al-Safadi L.A. (2014). Intervention Strategies for the Improvement of Students’ Academic Performance in Data Structure Course. Int. J. Inf. Educ. Technol..

[59] Coetzee D., Fox A., Hearst M.A., Hartmann B. Should your MOOC forum use a reputation system?. Proceedings of the 17th ACM conference on Computer Supported Cooperative Work & Social Computing.

[60] Burguillo J.C. (2010). Using game theory and competition-based learning to stimulate student motivation and performance. Comput. Educ..

[61] Stansfield M., McLellan E., Connolly T. (2004). Enhancing student performance in online learning and traditional face-to-face class delivery. J. Inf. Technol. Educ. Res..

[62] Zhang H., Almeroth K., Knight A., Bulger M., Mayer R. Moodog: Tracking students’ online learning activities. Proceedings of the EdMedia+ Innovate Learning. Association for the Advancement of Computing in Education (AACE).

[63] Kim J., Glassman E.L., Monroy-Hernández A., Morris M.R. RIMES: Embedding interactive multimedia exercises in lecture videos. Proceedings of the 33rd Annual ACM Conference on Human Factors in Computing Systems.

[64] Kim J., Guo P.J., Seaton D.T., Mitros P., Gajos K.Z., Miller R.C. Understanding in-video dropouts and interaction peaks inonline lecture videos. Proceedings of the first ACM conference on Learning@ scale conference.

[65] Park J.H., Choi H.J. (2009). Factors influencing adult learners’ decision to drop out or persist in online learning. J. Educ. Technol. Soc..

[66] Wen M., Yang D., Rose C. (2014). Sentiment Analysis in MOOC Discussion Forums: What Does It Tell Us? Educational Data Mining 2014. http://citeseerx.ist.psu.edu/viewdoc/download?doi=10.1.1.660.5804&rep=rep1&type=pdf.

[67] Ramesh A., Goldwasser D., Huang B., Daume H., Getoor L. Understanding MOOC discussion forums using seeded LDA. Proceedings of the Ninth Workshop on Innovative Use of NLP for Building Educational Applications.

[68] Kabakchieva D. (2013). Predicting student performance by using data mining methods for classification. Cybern. Inf. Technol..

[69] Balakrishnan G., Coetzee D. (2013). Predicting student retention in massive open online courses using hidden markov models. Electr. Eng. Comput. Sci. Univ. Calif. Berkeley.

